# Supplementary material for: Effects of acidification on nitrification and associated nitrous oxide emission in estuarine and coastal waters
Source: Nat Commun. 2023 Mar 13;14:1380. doi: 10.1038/s41467-023-37104-9 (PMC10011576; doi:10.1038/s41467-023-37104-9)
Supplement: Supplementary file 1 — Supplementary Information [file 41467_2023_37104_MOESM1_ESM.pdf]

Supplementary Materials for

Effects of acidification on nitrification and associated nitrous oxide emission in estuarine and coastal waters

Jie Zhou[1]†, Yanling Zheng[1,2,3,4]†*, Lijun Hou[1]*, Zhirui An[1], Feiyang Chen[1], Bolin Liu[1], Li Wu[2,3], Lin Qi[2,3], Hongpo Dong[1], Ping Han[2,3,4], Guoyu Yin[2,3,4], Xia Liang[1], Yi Yang[2,3,4], Xiaofei Li[1], Dengzhou Gao[1], Ye Li[2,3,4], Zhanfei Liu[5], Richard Bellerby[6], Min Liu[2,3,4]*

[1]State Key Laboratory of Estuarine and Coastal Research, Yangtze Delta Estuarine Wetland Ecosystem Observation and Research Station, East China Normal University, Shanghai 200241, China
[2]School of Geographic Sciences, East China Normal University, Shanghai 200241, China
[3]Key Laboratory of Geographic Information Science (Ministry of Education), East China Normal University, Shanghai 200241, China
[4]Key Laboratory of Spatial-temporal Big Data Analysis and Application of Natural Resources in Megacities, Ministry of Natural Resources, Shanghai 200241, China
[5]The University of Texas at Austin Marine Science Institute, Port Aransas, Texas 78373, USA
[6]Norwegian Institute for Water Research, Thormøhlensgt 53D, 5006 Bergen, Norway

†These authors contributed equally to this work.

*Corresponding authors. E-mail: ljhou@sklec.ecnu.edu.cn (L.J.H.); mliu@geo.ecnu. edu.cn (M.L.); ylzheng@geo.ecnu.edu.cn (Y.L.Z.)

This PDF file includes:

Supplementary Methods
Supplementary Text 1 to 3
Supplementary Figures 1 to 17
Supplementary Tables 1 to 10
Supplementary References

Supplementary Methods

DNA extraction, pyrosequencing, and quantitative PCR

Total genomic DNA was extracted using a PowerWater™ DNA Isolation Kit (MOBIO, USA) based on the manufacturer's protocol and preserved at -80 °C. Triplicate qualified DNA extractions from each sample were pooled for further pyrosequencing and qPCR analysis. Illumina MiSeq sequencing was conducted based on the V4-V5 hypervariable region of the bacterial 16S rRNA gene with primers 515F (5′-GTGCCAGCMGCCGCGGTAA-3′) and 909R (5′-CCCCGYCAATTCMTT TRAGT-3′)[1] and the V3-V4 hypervariable region of the archaeal 16S rRNA gene with primers 349F (5′-GYGCASCAGKCGMGAAW-3′) and 806R (5′-GGACTACVSGGGTATCTAAT-3′)[2]. In addition, the modified V4 16S rRNA gene primers 515F-modified (5′-GTGYCAGCMGCCGCGGT AA-3′)[3] and 806R-modified (5′-GGACTACNVGGGTWTCTAAT-3′)[4] were further used for the simultaneous detection, sequencing, and examination of the relative abundances of both bacteria and archaea within the same datasets. PCR amplicons were purified using Agencourt AMPure XP (Beckman Coulter, Brea, CA), quantified (Qubit2.0 DNA, Life Technologies), and pooled with an equimolar concentration, then applied to an Illumina Miseq system (Illumina, USA) for sequencing. Sequences were processed and the taxonomy was assigned using Qiime 1.9.0[5]. Briefly, all sequence reads were trimmed and assigned to each sample based on their barcodes. Chimera sequences were removed using the Uchime algorithm[6] and high-quality sequences (average base quality score>30, length>150 bp, and without ambiguous base 'N') were used for downstream analysis. Operational taxonomic units (OTUs) were classified using a 97% identity threshold. Taxonomy assignment was conducted based on Ribosomal Database Project (RDP) classifier at a minimum confidence level of 80%[7].

Real-time quantitative PCR (qPCR) was performed with ABI 7500 (Applied Biosystems, Canada) to determine the abundance of bacterial *amoA* gene, archaeal *amoA* gene, comammox *amoA* gene, and *Nitrospira* 16S rRNA gene (Supplementary Table 10). Corroboration of gene expression in metatranscriptomic method was also conducted via qPCR with complementary DNA (cDNA, which was obtained from the triplicate mRNA using Superscript Double-Stranded cDNA Synthesis kit of Invirtrogen and a random hexamer primer) as template. Prior to qPCR, all DNA and cDNA samples were normalized to a concentration of 10 ng μl^{-1}. The quantitative standard for each gene was constructed as described previously[1]. qPCRs were performed in triplicate reactions with corresponding primers under thermocycling conditions as given in Supplementary Table 10. The 25-μl qPCR mixtures contained 12.5 μl of Maxima SYBR Green/Rox qPCR Master Mix (Fermentas, Lithuania), 1 μl of each primer (10 μM), and 1 μl of template DNA or cDNA. To lower the possibility of over-estimation, melting curves and gel electrophoreses were both performed. In addition, in all qPCR assays, three negative controls without any DNA template additions were also conducted in all the amplification reactions. Gene abundance was calculated based on the quantitative standard curve and converted to copies per ml. In this study, qPCR results with amplification efficiency between 95-105% and correlation coefficient above 0.98 were employed.

Natural isotopic signatures of N_2O

To identify the pathway of N_2O production, natural-abundance isotope analyses were performed. Briefly, headspace gas was collected from the reactors using gas-tight syringes for analyses of N_2O isotopomer ratios ($\delta^{15}N^{bulk}$, $\delta^{15}N^{\alpha}$, and $\delta^{18}O$) using isotope ratio mass spectrometry (IRMS, Delta V Plus, Thermo Fisher Scientific, Bremen, Germany). The typical analytical precisions for $\delta^{15}N^{bulk}$, $\delta^{15}N^{\alpha}$ and $\delta^{18}O$ were 0.5‰, 0.9‰ and 0.6‰, respectively. The ^{15}N-site preference (*SP*) is the difference of $^{15}N/^{14}N$ between the central ($\delta^{15}N^{\alpha}$) and terminal ($\delta^{15}N^{\beta}$) N positions in N_2O[8], and it was calculated as below:

$$\delta^{15}N^{\text{bulk}} = (\delta^{15}N^{\alpha} + \delta^{15}N^{\beta})/2 \quad (1)$$
$$SP = \delta^{15}N^{\alpha} - \delta^{15}N^{\beta} \quad (2)$$
The scrambling factor in the ion source of this mass spectrometer was determined using a series of $^{\beta}$N-labelled N_2O, and SP-N_2O was further corrected[9]. N_2O working standard gas produced by Air Liquide America, Specialty Gases LLC was used to determine calibration. Characteristic *SP* values of 33‰ for NH_2OH oxidation and 0‰ for NO_2^- reduction, which were estimated in pure cultures[10,11], were used to estimate the contribution of each process, assuming that each process is linearly proportional to the *SP* value using the following equations[11].
$$F_{\text{NO}_2\text{- reduction}}(\%) = \frac{(SP - \text{SP}_{\text{NH}_2\text{OH oxidation}})}{(\text{SP}_{\text{NO}_2\text{- reduction}} - \text{SP}_{\text{NH}_2\text{OH oxidation}})} \times 100\% \quad (3)$$
$$F_{\text{NH}_2\text{OH oxidation}}(\%) = 100\% - F_{\text{NO}_2\text{- reduction}} \quad (4)$$
where $F_{\text{NO}_2\text{- reduction}}$ and $F_{\text{NH}_2\text{OH oxidation}}$ denote the contribution of NO_2^- reduction and NH_2OH oxidation to the total N_2O production.
On this basis, the calculated *SP* values in our continuous-flow manipulation systems were from 28.6 to 33.8. Thus, it was estimated that 86.7-100% of the released N_2O was produced via NH_2OH oxidation, whereas the contribution of NO_2^- reduction was 0-13.3% (Supplementary Table 4).

Supplementary Text

Supplementary Text 1. Using the estimated global estuarine area of 1.025×10^6 km^2 [12] and the median water-air N_2O flux of about 21.6 μmol m^{-2} d^{-1} in estuarine waters, it was estimated that an approximately 0.23 Tg N_2O-N was released into the air from the global estuarine waters per year (Supplementary Table 5a and 5b). Likewise, using the estimated global continental shelf area of 2.72×10^7 km^2 [13] and the median water-air N_2O flux of about 3.1 μmol m^{-2} d^{-1} in continental shelf waters, an estimated amount of 0.86 Tg N_2O-N was released from continental shelves per year. Totally, the estimated global N_2O flux in estuarine and coastal continental shelves was about 1.09 Tg N_2O-N yr^{-1} (Supplementary Table 5b). Assumed that nitrifiers contributed to half of these N_2O emissions[12,14], nitrification N_2O emission in these ecosystems would increase by 0.05-0.15 Tg N_2O-N yr^{-1} in response to a decrease of 0.21 pH units.

Supplementary Text 2. Positive feedback was reported for nitrogen-fixing cyanobacteria under higher pCO_2 conditions[15,16], which is due to the under-saturation of ribulose bisphosphate carboxylase oxidase (RuBisCO), the main enzyme that is responsible for CO_2 fixation in the Calvin cycle, under the current environmental CO_2 concentrations[17]. Similarly, ammonia-oxidizing bacteria (AOB), including *Nitrosomonas*, *Nitrosospira*, and *Nitrosococcus* also assimilate CO_2 through the Calvin cycle[18,19]. Therefore, the ongoing increase in dissolved CO_2 in the estuarine and coastal water columns might also benefit them. In the present study, a significant down-regulation of gene expression associated with the carbon-concentrating mechanism (ccm) that saturates the carboxylating enzyme, Rubisco, was observed under acidified conditions, suggesting a reduced energetic requirement for CO_2 enrichment[19]. However, the saved energy seemed minor when compared with the disturbances caused by acidification, as significant negative responses were observed under acidified conditions.

Supplementary Text 3. Transcripts of genes encoding the ribulose-bisphosphate carboxylase (RBCL) and phosphoglycerate kinase (PGK) of the Calvin cycle, 2-oxoglutarate/2-oxoacid ferredoxin oxidoreductase (KOR), aconitate hydratase (ACN), ATP-citrate lyase ACO), malate dehydrogenase (MDH), fumarate hydratase (FUM), succinate dehydrogenase/fumarate reductase (SDH) of the rTCA cycle were significantly down-regulated (52-95%) under acidified conditions (Fig. 4b and 4c).

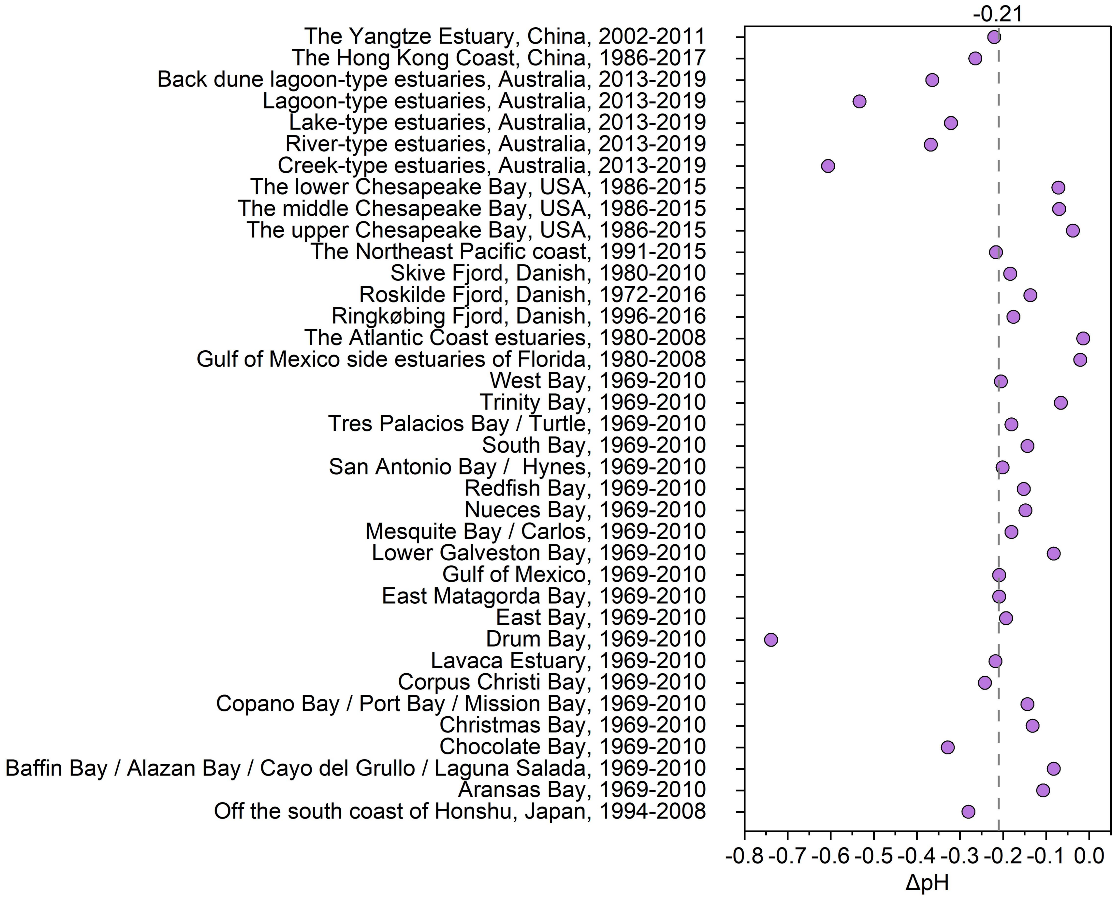

Supplementary Fig. 1. Observed pH decreases (ΔpH) in estuarine and coastal waters across the world over the past several decades. The dotted line shows that there was an average reduction of about 0.21 pH units. These data are obtained from the references[20-28]. Source data are provided as a source data file.

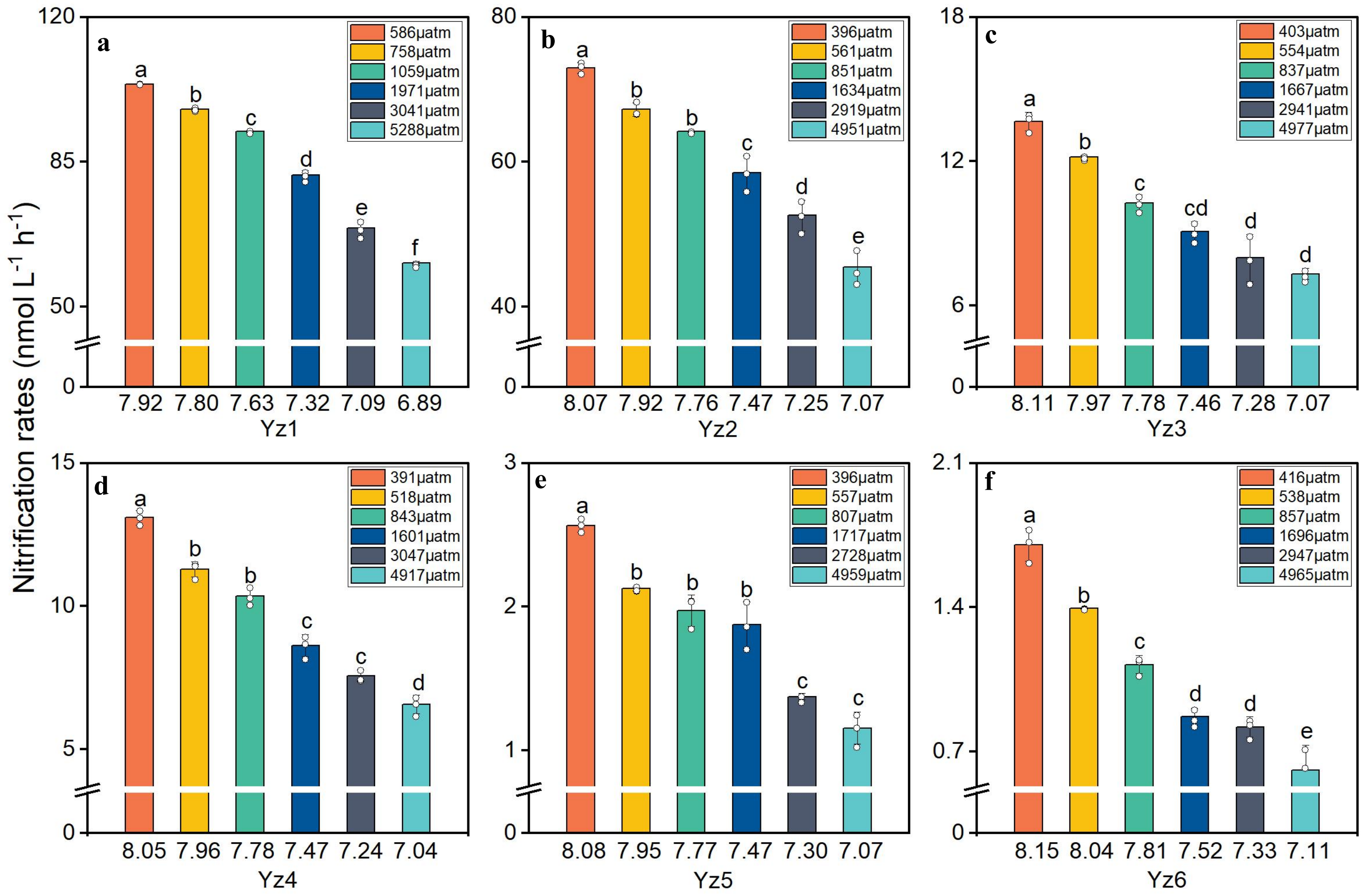

Supplementary Fig. 2. Effects of simulated aquatic acidification on nitrification in the Yangtze Estuary and adjacent coastal waters. (**a**) Yz1; (**b**) Yz2; (**c**) Yz3; (**d**) Yz4; (**e**) Yz5; (**f**) Yz6. Error bars denote SD (n=3 biologically independent samples), and dots are corresponding data points of the replicates. Different lowercase letters (a, b, c, d, e and f) above the columns indicate significant differences (P<0.05). Significant differences were determined via one-way analysis of variance (ANOVA). Source data are provided as a source data file.

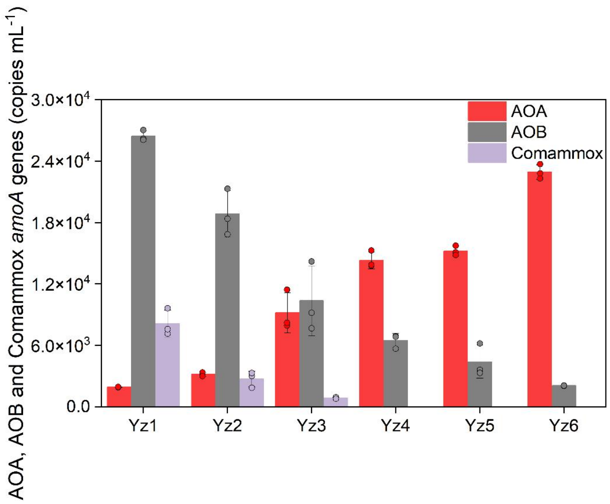

Supplementary Fig. 3. Abundance of ammonia-oxidizing archaea (AOA), ammonia-oxidizing bacteria (AOB) and comammox *amoA* gene in the six sites of the Yangtze Estuary and adjacent coastal waters. Error bars denote SD (n=3 biologically independent samples), and dots are corresponding data points of the replicates. Source data are provided as a source data file.

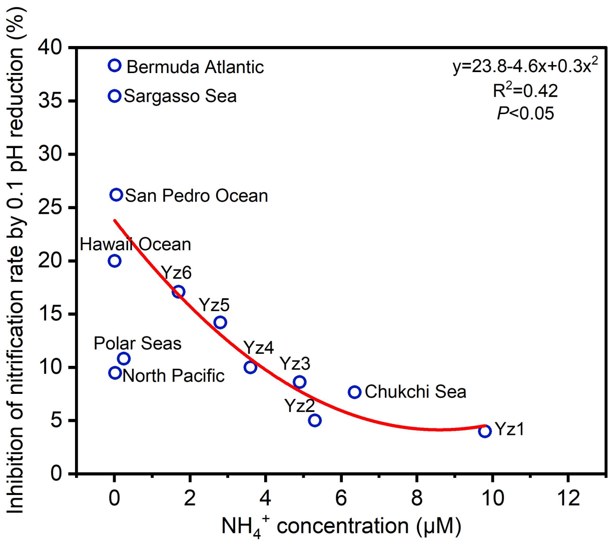

Supplementary Fig. 4. Relationship between the NH_4^+ concentration and the inhibition of nitrification rate by acidification in the Yangtze Estuarine and coastal waters (Yz1-Yz6) and other seawaters[17,29-31]. Source data are provided as a source data file.

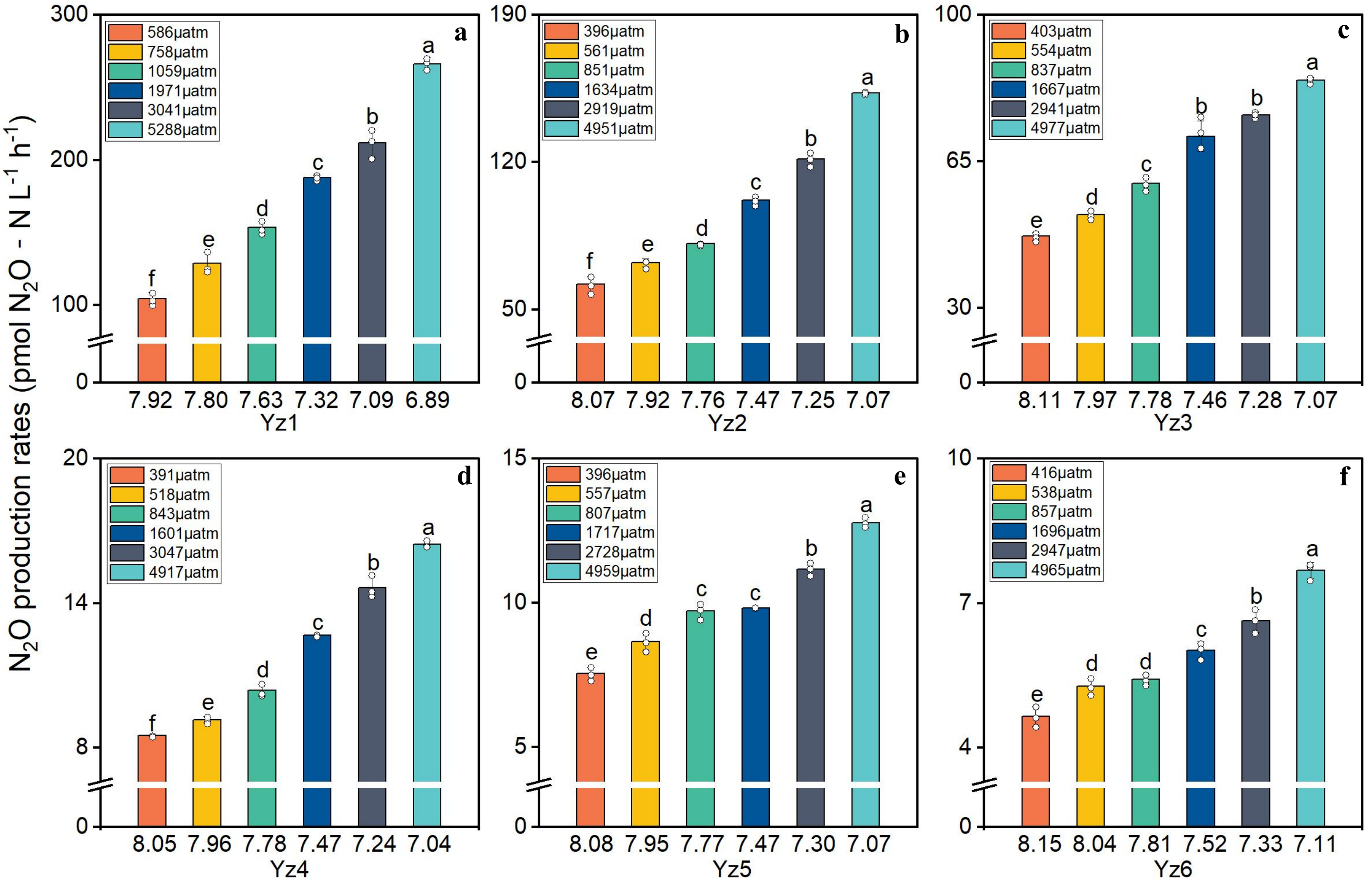

Supplementary Fig. 5. Effects of simulated aquatic acidification N_2O production rates in the Yangtze Estuary and adjacent coastal waters. (**a**) Yz1; (**b**) Yz2; (**c**) Yz3; (**d**) Yz4; (**e**) Yz5; (**f**) Yz6. Error bars denote SD (n=3 biologically independent samples), and dots are corresponding data points of the replicates. Different lowercase letters (a, b, c, d, e and f) above the columns indicate significant differences (P<0.05). Significant differences were determined via one-way analysis of variance (ANOVA). Source data are provided as a source data file.

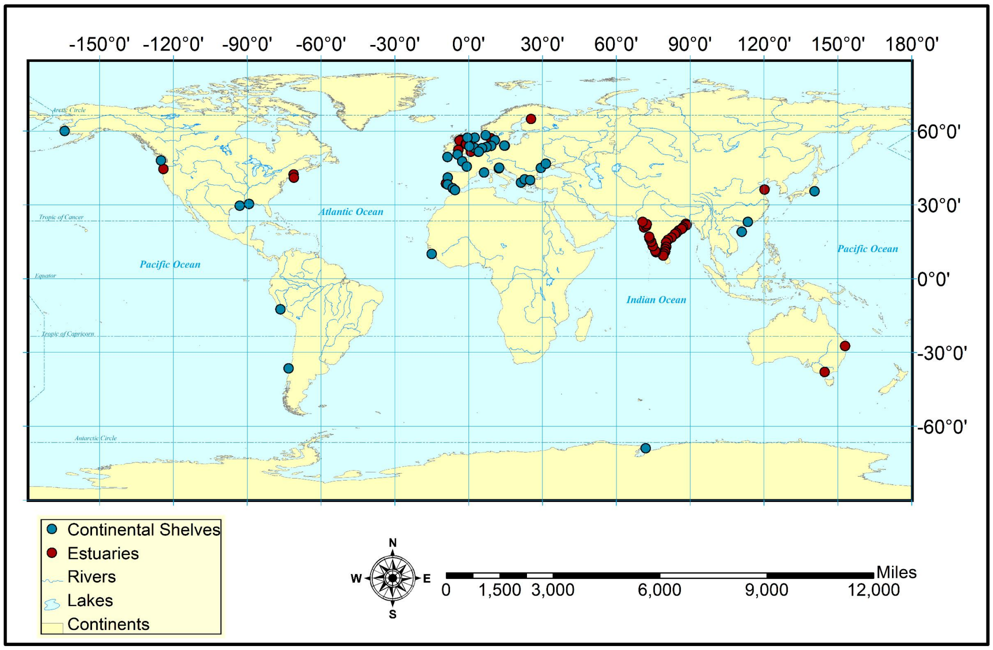

Supplementary Fig. 6. Locations of N_2O flux measurements worldwide. Specific N_2O flux data are given in Supplementary Table 5.

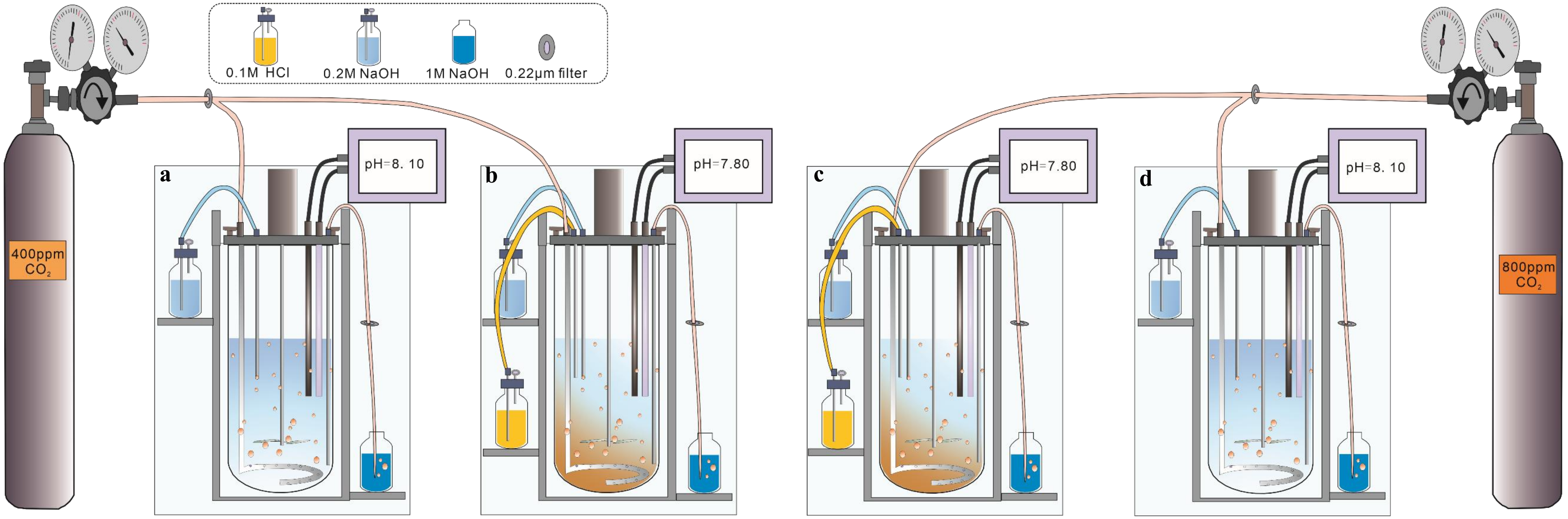

Supplementary Fig. 7. The constructed continuous airflow manipulating equipments to decouple the effects of elevated *p*CO_2 and reduced pH on nitrifiers under acidified conditions. pH and *p*CO_2 in these four groups were maintained around: (**a**) 8.1/400 µatm; (**b**) 7.8/400 µatm; (**c**) 7.8/800 µatm; (**d**) 8.1/800 µatm. The carbonate chemistry was manipulated by bubbling water samples with 0.22 µm-filtered CO_2 adjusted air while adjusting pH with sterile acid (0.1 M HCl) or base (0.2 M NaOH) solution through the reactor's acid-base automatic regulation system. The inlet air:CO_2 bubbles were gently dispersed in the water body by two stirrers which were installed at the bottom and above the inlet airflow (30 rpm), to make the water system uniform. The acid gas in the outlet airflow was absorbed by 1 M NaOH or collected with gas sampling bags (Teflon®FEP, DuPont) at the beginning of rate measurements after equilibrium. The temperature was maintained at room temperature (25 °C) by an automatic heating plate and cold circulating water bath. During the incubation, DO, temperature, and pH were recorded in real-time through the electrodes equipped with bioreactors. The incubations were conducted in dark by covering the bioreactors with opaque paper.

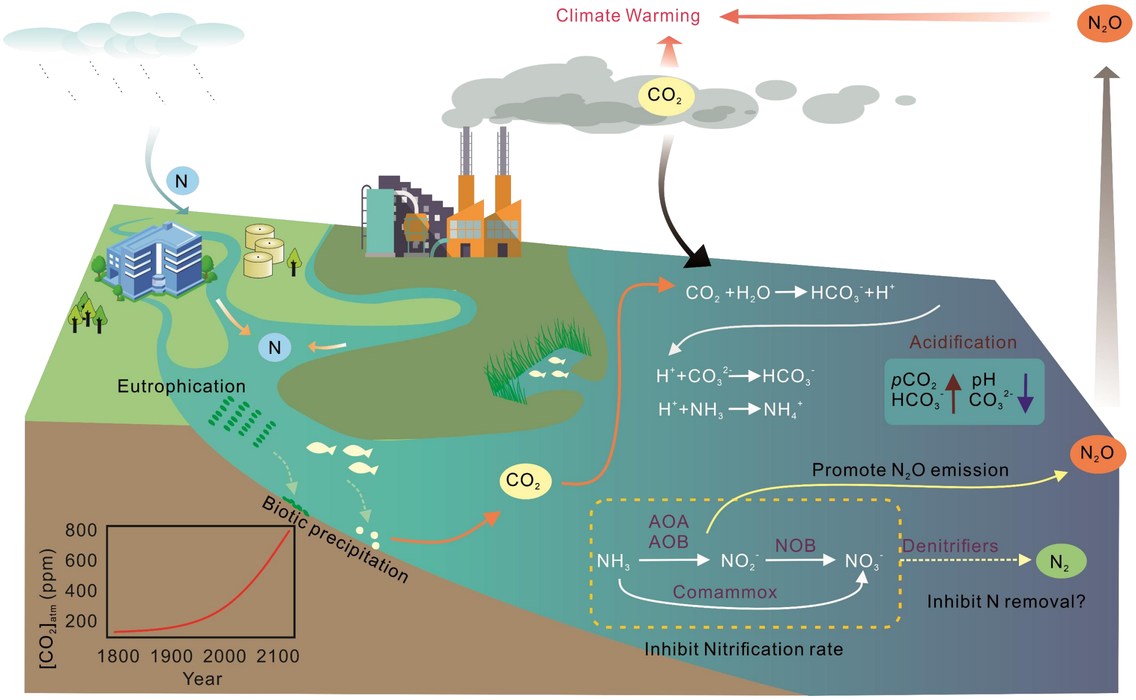

Supplementary Fig. 8. Effects of acidification on nitrification process in estuarine and coastal ecosystems and potential feedback to climate warming. Acidification inhibits nitrification rate, increases N_2O emissions, and accelerates climate warming.

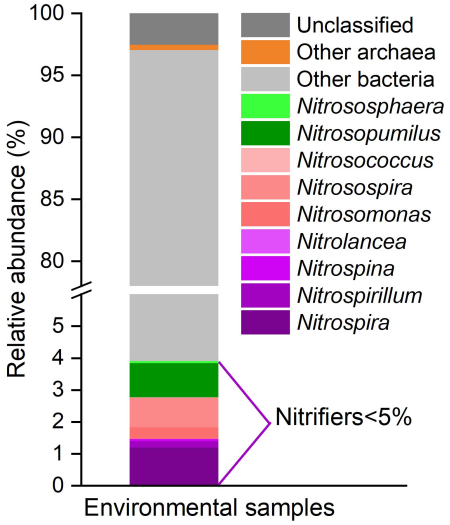

Supplementary Fig. 9. Relative abundance of nitrifiers in the collected water samples from site Yz3. The relative abundance data were acquired based on the 16S rRNA gene sequencing with universal primers [515F-modified[3]/806R-modified[4]] capable of detecting both bacteria and archaea within the same sequencing libraries. Source data are provided as a source data file.

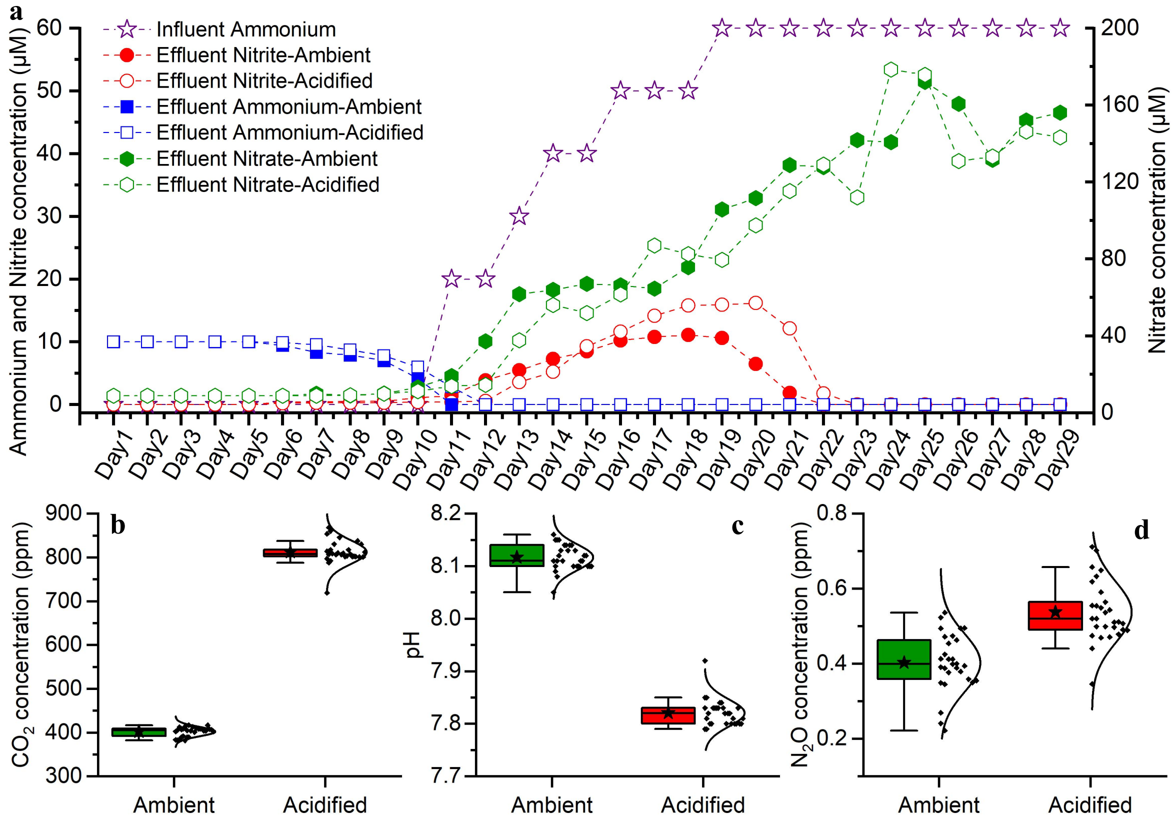

Supplementary Fig. 10. Performance of the continuous flow environmental simulation systems. (**a**) The performance as measured by NH_4^+, NO_3^-, and NO_2^- concentrations in the effluent of the ambient control and acidified treatments in the simulation systems. (**b**) CO_2 concentrations in the ambient control (with an average value of 400 ppm) and acidified treatments (with an average value of 800 ppm) during the incubation period. (**c**) The pH conditions in the ambient control (with an average value of 8.1) and acidified treatments (with an average value of 7.8) during the incubation period. (**d**) The concentration of N_2O in the outlet gas of the ambient control and acidified treatments during the incubation period in the simulation systems. Horizontal lines in the box charts indicate the medium, stars represent the mean. The boxes give the 25th and 75th percentiles, the whiskers show range from 5th to 95th percentiles, and the curves show the distribution of the values. At the beginning of the long-term incubation, NH_4^+ concentration of water samples in the manipulation systems was supplemented to a final concentration of approximately 10 µM. After about half of the NH_4^+ was consumed (day 10), filter-sterilized site water with proper NH_4^+ concentrations (gradually increased from 20 µM to 60 µM with the increase of NH_4^+ consumption rate) were used as culture medium and supplied at a flow rate of 1 L day^{-1} to all reactors. Since the influent culture medium was continuously added to the manipulation systems drop by drop, the added NH_4^+ can be rapidly consumed and thus the NH_4^+ concentration in the effluent was close to zero. n=29 independent samples at different sampling times. Source data are provided as a source data file.

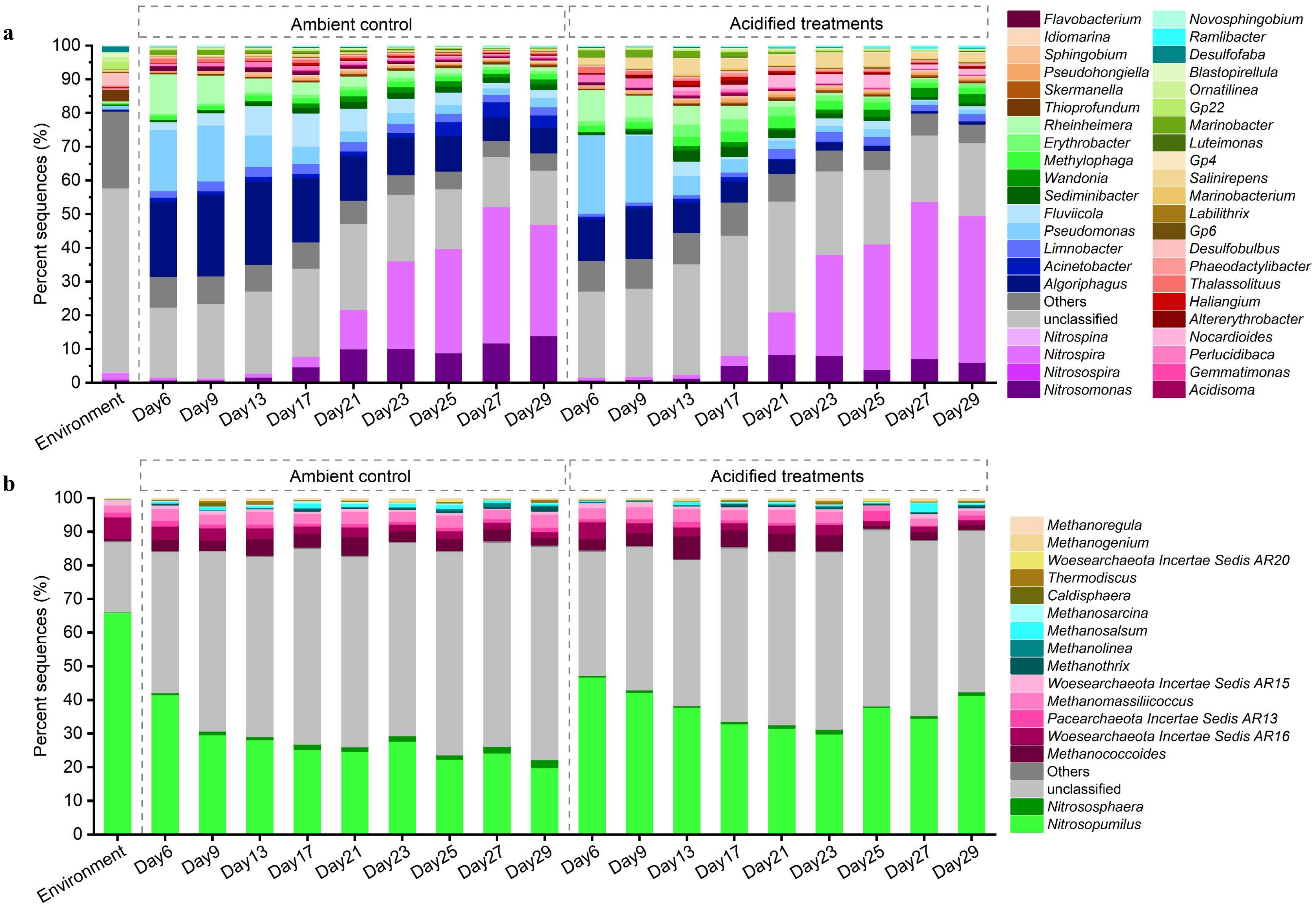

Supplementary Fig. 11. Bacterial (a) and archaeal (b) community compositions in the ambient control (pCO$_2$=400 μatm, pH=8.1) and acidified treatments (pCO$_2$=800 μatm, pH=7.8) in the continuous flow simulation systems during the incubation period and the original near-bottom water samples collected from site Yz3 based on 16S rRNA gene sequencing. Bacterial and archaeal community compositions were acquired based on 16S rRNA gene sequencing with bacterial and archaeal primers, respectively. Source data are provided as a source data file.

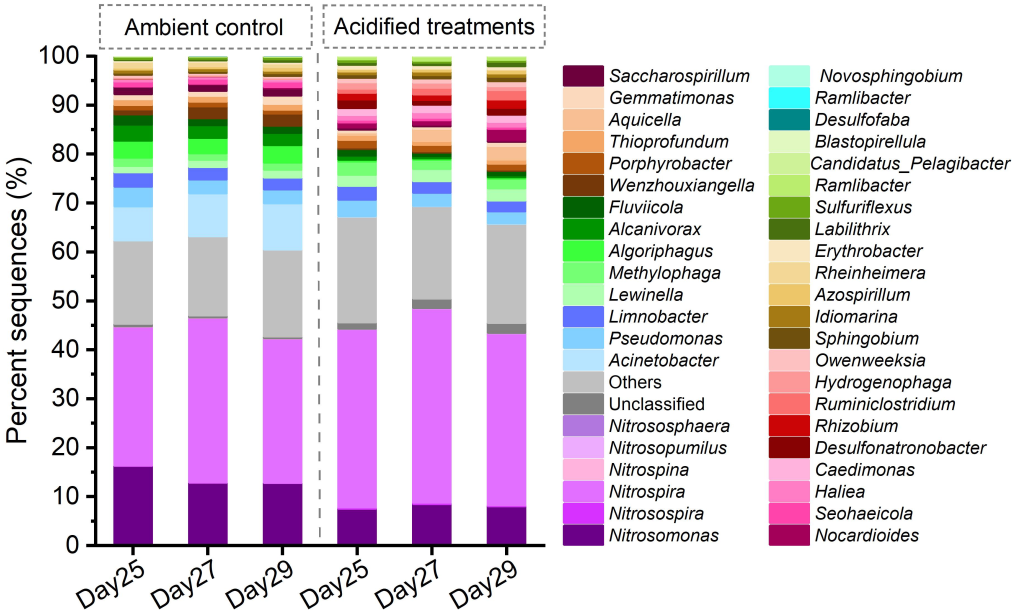

Supplementary Fig. 12. Microbial community compositions of the enrichment culture at the ambient controls (*p*CO_2=400 µatm; pH=8.1) and acidified treatments (*p*CO_2=800 µatm; pH=7.8) in the continuous flow simulation systems. The relative abundance data were acquired based on the 16S rRNA gene sequencing with universal primers capable of detecting both bacteria and archaea within the same sequencing libraries. Nitrifying communities averagely account for 44.6% and 45.5% in the ambient controls and acidified treatments, respectively. Source data are provided as a source data file.

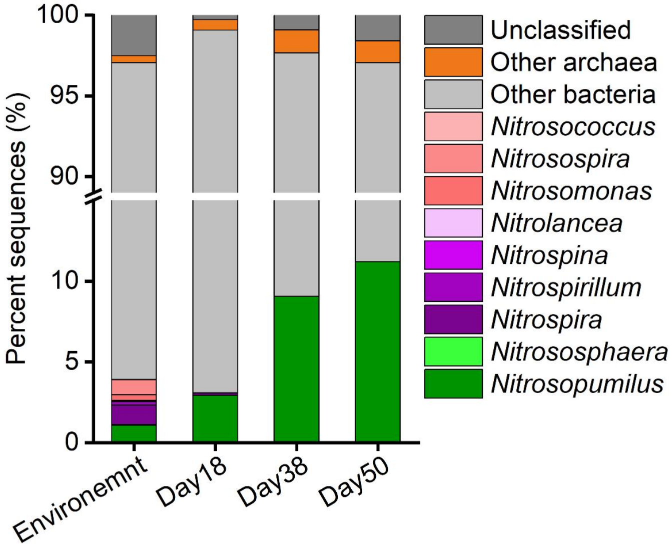

Supplementary Fig. 13. Microbial community compositions of the AOA enrichment culture. The relative abundance data were acquired based on the 16S rRNA gene sequencing with universal primers capable of detecting both bacteria and archaea within the same sequencing libraries. Source data are provided as a source data file.

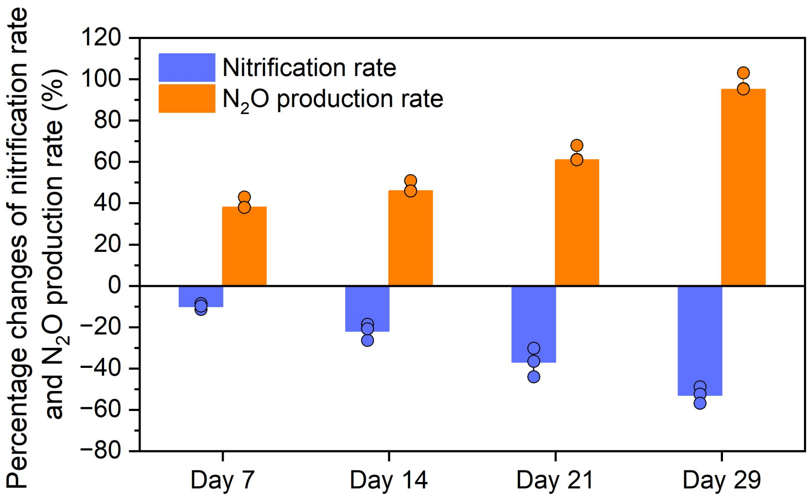

Supplementary Fig. 14. Effect of aquatic acidification on nitrification rate and associated N_2O emission during the long-term acidification experiments (800 µatm/pH 7.8). Data show the percentage changes nitrification rate and N_2O emission in the acidified treatments compared to the ambient control. DO was maintained above 6.5 mg L^{-1} during the incubation. Error bars denote SD (n=3 biologically independent samples), and dots are corresponding data points of the replicates. Source data are provided as a source data file.

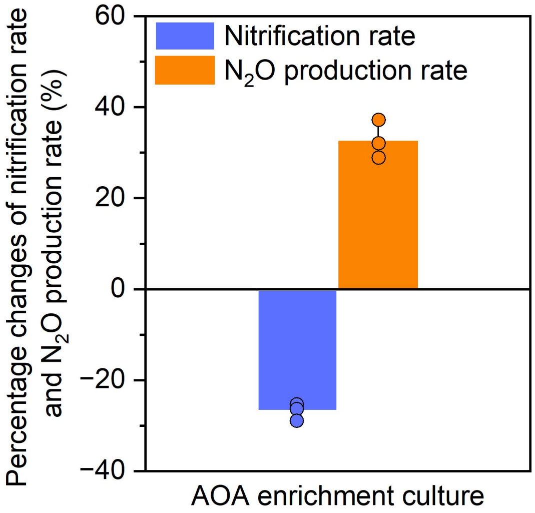

Supplementary Fig. 15. Effect of aquatic acidification on nitrification rate and associated N_2O emission of the AOA enrichment culture. Data show the percentage changes nitrification rate and N_2O emission in the acidified treatments (800 µatm/pH 7.8) compared to the ambient control (400 µatm/pH 8.1). Error bars denote SD (n=3 biologically independent samples), and dots are corresponding data points of the replicates. Source data are provided as a source data file.

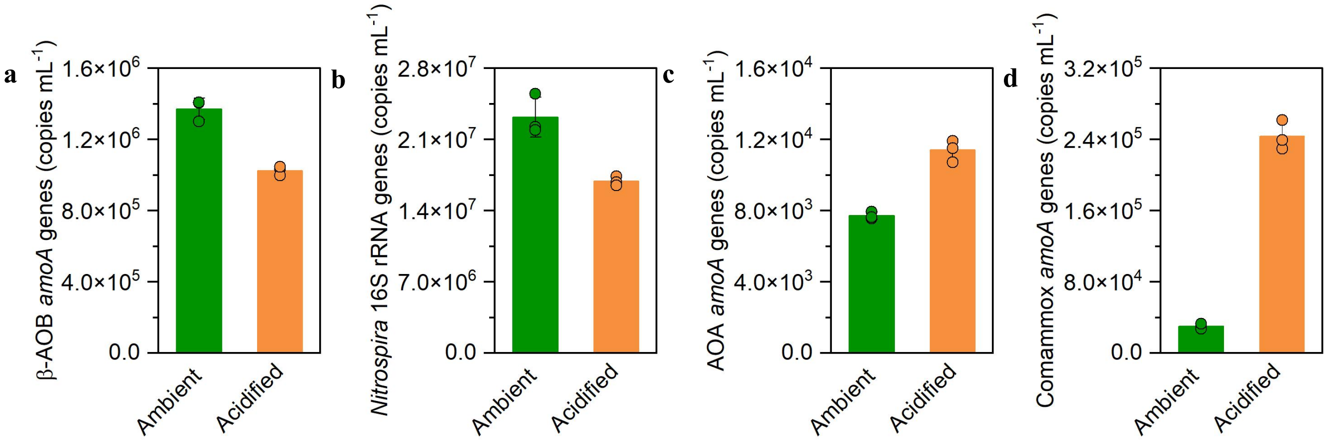

Supplementary Fig. 16. Abundance of ammonia-oxidizing bacteria (AOB, dominated by *Nitrosomonas*) (**a**), *Nitrospira* (**b**), ammonia-oxidizing archaea (AOA, dominated by *Nitrosopumilus*) (**c**), and comammox (**d**) in the ambient control and acidified treatments at the end of the long-term acidification experiments. The abundance of comammox *amoA* gene was equal to that of comammox clade A *amoA* gene, as comammox clade B *amoA* gene was not detected. Error bars denote SD (n=3 biologically independent samples), and dots are corresponding data points of the replicates. Source data are provided as a source data file.

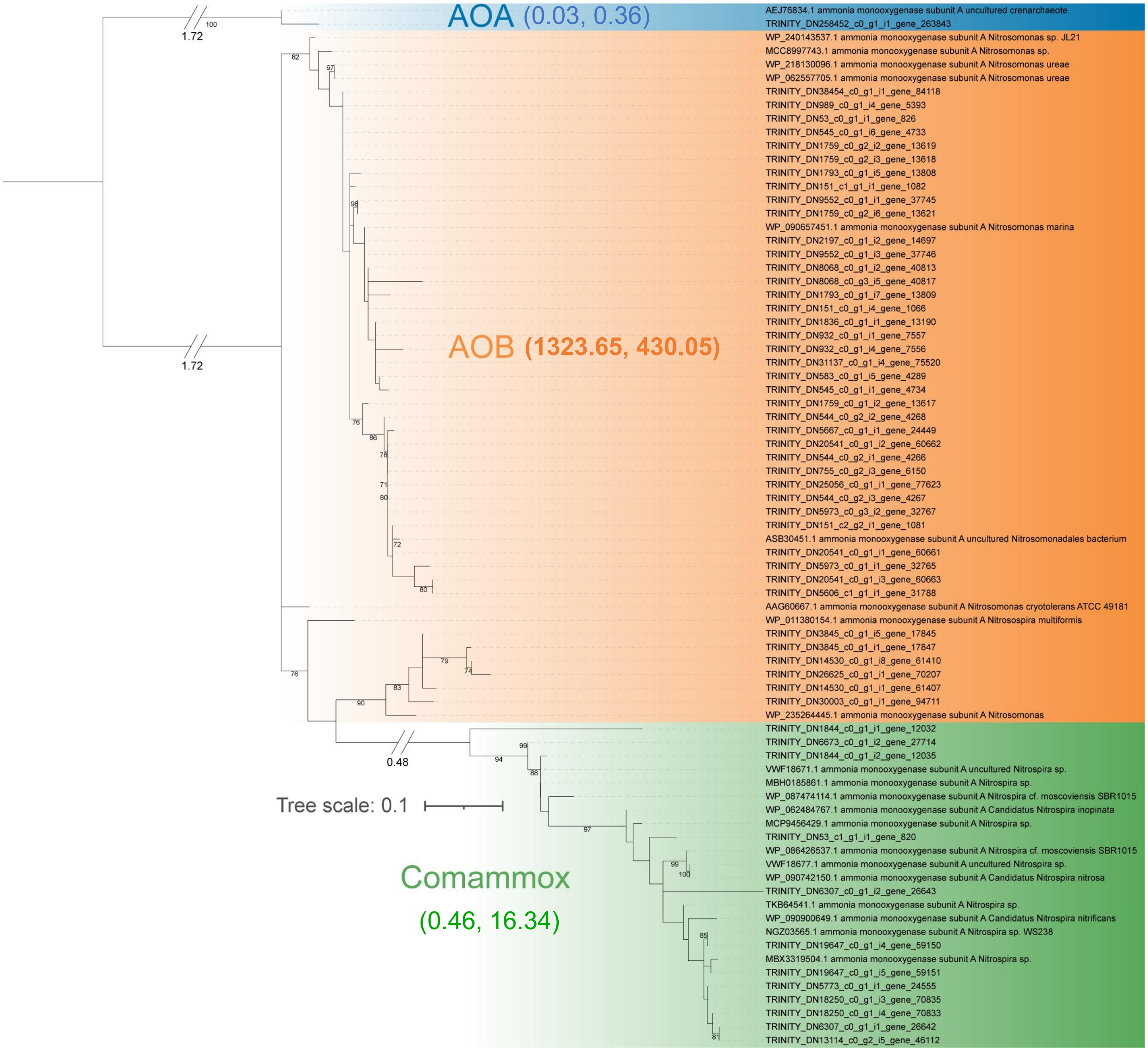

Supplementary Fig. 17. Maximum-likelihood phylogenetic tree of ammonia monooxygenase subunit A (amoA) based on metatranscriptome data. Numbers near nodes are percentages of bootstrap values based on 1000 re-samplings (higher than 70% are shown). Sequences obtained in this study are shown in boldface. GenBank accession numbers are shown for reference sequences. The scale bar refers to a phylogenetic distance of 0.1 amino substitutions per site. Numbers near the breaks indicate branch lengths. AOA, ammonia-oxidizing archaea; AOB, ammonia-oxidizing bacteria; Comammox, complete ammonia oxidizers. Numbers in parentheses following each group indicate the TPM values of *amoA* gene transcripts in the ambient control (left) and acidified treatments (right).

Supplementary Table 1. Environmental parameters in near-bottom waters of sampling sites.

Sites	pH	Salinity	DO mg/L)	NO_3^- (μM)	NO_2^- (μM)	NH_4^+ (μM)	Depth (m)
Yz1	7.92	2.93	8.95	100.5	0.5	9.8	11
Yz2	8.07	18.17	8.78	69.6	0.6	5.3	15
Yz3	8.11	20.45	9.86	60.2	0.8	4.9	9
Yz4	8.05	31.28	9.15	26.3	0.5	3.6	18
Yz5	8.08	32.99	8.50	10.2	0.4	2.8	30
Yz6	8.15	33.85	8.30	8.6	0.1	1.7	61

All parameters were measured in triplicate.

Supplementary Table 2. pH, pCO_2, and dissolved oxygen concentration (DO) during the acidification experiments.

Sites	pH	Δ pH	pCO_2 (μatm)	Δ pCO_2	DO (mg/L)
Yz1	*7.92 ± 0.01*	*0*	*586 ± 12*	*0*	*8.01 ± 0.04*
	7.80 ± 0.01	-0.12	*758 ± 16*	172	*7.99 ± 0.04*
	7.63 ± 0.02	-0.29	1059 ± 38	472	7.97 ± 0.03
	7.32 ± 0.02	-0.60	1971 ± 77	1385	7.99 ± 0.05
	7.09 ± 0.01	-0.83	3041 ± 49	2455	8.05 ± 0.05
	6.89 ± 0.01	-1.03	5288 ± 77	4701	8.04 ± 0.01
YZ2	*8.07 ± 0.01*	*0*	*396 ± 9*	*0*	*8.04 ± 0.01*
	7.92 ± 0.02	-0.15	*561 ± 21*	166	7.98 ± 0.01
	7.76 ± 0.04	-0.31	851 ± 91	456	8.03 ± 0.03
	7.47 ± 0.02	-0.60	1634 ± 95	1238	8.08 ± 0.02
	7.25 ± 0.02	-0.82	2919 ± 128	2524	8.02 ± 0.02
	7.07 ± 0.03	-1.00	4951 ± 342	4555	8.05 ± 0.03
Yz3	*8.11 ± 0.01*	*0*	*403 ± 5*	*0*	*8.00 ± 0.05*
	7.97 ± 0.02	-0.13	*554 ± 20*	151	7.97 ± 0.02
	7.78 ± 0.01	-0.32	837 ± 26	434	7.98 ± 0.03
	7.46 ± 0.01	-0.64	1667 ± 30	1264	8.01 ± 0.05
	7.28 ± 0.02	-0.83	2941 ± 178	2538	8.07 ± 0.06
	7.07 ± 0.02	-1.03	4977 ± 226	4574	8.01 ± 0.03
Yz4	*8.05 ± 0.01*	*0*	*391 ± 6*	*0*	*8.01 ± 0.03*
	7.96 ± 0.03	-0.10	518 ± 32	127	7.99 ± 0.01
	7.78 ± 0.01	-0.27	843 ± 11	452	8.03 ± 0.04
	7.47 ± 0.01	-0.58	1601 ± 26	1210	8.01 ± 0.04
	7.24 ± 0.02	-0.81	3047 ± 128	2656	8.00 ± 0.03
	7.04 ± 0.01	-1.01	4917 ± 145	4525	8.02 ± 0.05
Yz5	*8.08 ± 0.01*	*0*	*396 ± 9*	*0*	*7.99 ± 0.04*
	7.95 ± 0.01	-0.13	557 ± 7	160	8.02 ± 0.03
	7.77 ± 0.02	-0.31	807 ± 32	411	8.11 ± 0.07
	7.47 ± 0.01	-0.61	1717 ± 46	1320	8.03 ± 0.06
	7.30 ± 0.01	-0.78	2728 ± 83	2332	8.12 ± 0.05
	7.07 ± 0.02	-1.01	4959 ± 279	4563	8.02 ± 0.04
YZ6	*8.15 ± 0.01*	*0*	*416 ± 5*	*0*	*8.03 ± 0.02*
	8.04 ± 0.01	-0.11	*538 ± 13*	122	8.00 ± 0.03
	7.81 ± 0.01	-0.34	857 ± 13	442	8.03 ± 0.04
	7.52 ± 0.02	-0.64	1696 ± 104	1280	8.11 ± 0.04
	7.33 ± 0.01	-0.83	2947 ± 49	2531	8.13 ± 0.02
	7.11 ± 0.01	-1.05	4965 ± 88	4550	8.04 ± 0.03

Control groups are shown in italics. ΔpH and ΔpCO_2 correspond to the changes of pH and pCO_2 before and after acidification, respectively. Errors denote SD (n=3 biologically independent samples).

Supplementary Table 3. Equations for the fitted curves of percentage changes of nitrification and N_2O emission rates with ΔpH values in Fig. 2.

	Nitrification	R^2	*P* value	N_2O emission	R^2	*P* value
Yz1	$y=-0.857+30.962x-8.841x^2$	0.99	*<0.001*	$y=4.237-102.632x+39.606x^2$	0.98	*0.001*
Yz2	$y=-2.169+27.325x-7.338x^2$	0.97	*0.002*	$y=9.345-38.965x+97.127x^2$	0.99	*<0.001*
Yz3	$y=-1.475+74.601x+30.438x^2$	0.99	*<0.001*	$y=0.681-72.399x+2.85x^2-$	0.99	*<0.001*
Yz4	$y=-2.953+73.439x+29.623x^2$	0.97	*0.003*	$y=0.027-73.562x+18.024x^2$	0.99	*<0.001*
YZ5	$y=-5.154+95.345x+53.794x^2$	0.96	*0.003*	$y=5.672-6.761x+54.98x^2$	0.90	*0.015*
YZ6	$y=-9.238+81.512x+29.735x^2$	0.98	*0.001*	$y=5.962-17.373x+36.049x^2$	0.96	*0.004*

Supplementary Table 4. Contribution of NO_2^- reduction and NH_2OH oxidation to the total N_2O production based on natural isotopic signatures of N_2O.

	Yz1	Yz2	Yz3	Yz4	Yz5	Yz6
SP	28.6±0.6	30.9±0.9	29.5±0.7	31.5±1.2	33.8±0.9	30.7±0.3
NH_2OH oxidation (%)	86.7	93.6	89.4	95.5	100.0	93.0
NO_2^- reduction (%)	13.3	6.4	10.6	4.5	0.0	7.0

Mean±SD (n=3 biologically independent samples).

Supplementary Table 5a. N_2O flux in estuarine waters and coastal continental shelves.

Location of sites	N_2O flux range	Mean flux	References
	μmol N_2O m^{-2} d^{-1}		
Estuarine waters			
The western Oder estuary, Germany	0.27-7.2	3.29	32
The Humber Estuary and the Tweed Estuary, UK		43.2	33
The Alsea Estuary, USA	1.2-17.3	5.52	34
The Scheldt River Estuary, France	~9.6-528	198.24	35
The Seine River Estuary, France	76.8-110.4	96.48	36
The Tagus Estuary, Portugal	-1-12	2.88	37
The Lendrup Vig Estuary Denmark	4.1-43.2	22.8	38
28 estuaries along the Indian coast	-0.01-14.21	1.97	39
The Child's River Estuary, USA	0-38.4	11.04	40
The Tamar River Estuary, UK	0-36	9.6	41
The Sacca di goro Estuary, Italy	-4.3-158.4	21.6	42
The Brisbane River Estuary, Australia	2.2-76.8	15.12	43
The Colne Estuary, UK	0-1339.2	31.2	44
The Temmesjoki Estuary, Finland		15.12	45
The Werribee Estuary, Australia	66-96	81.6	46
The Adyar River Estuary, India		21.6	47
The Pearl River Estuary, China	0-350	75	48
Coastal continental shelves			
The European Central Celtic Coast	-0.22-3.36	1.57	49
The Northern North Sea Coast	-0.34-3.52	1.59	49
The European Long Forties Coast	-0.2-3.94	1.87	49
The Southern North Sea Coast	0.66-4.52	2.59	49
Continental Shelf off Peru		39	50
Continental Shelf off Chile	-9.8-195	30.2	51
Continental Shelf of Bering Sea	-3.2-23.6	8.2	52
Continental shelf of southern Vancouver Island	-4.5-21.9	4.2	53
The Prydz Bay, Antarctica	0.76-4.6	2.4	54
Continental shelf of west Africa	0-0.35	0.17	55
Continental shelf of European		8.43	56
The north-eastern shelf of the Cádiz Gulf	0.4-22.5	9.35	57
The eastern shelf of the Cadiz Gulf	0.7-4.7	2.7	58
Shelf of Eastern Ionian Sea		0.1	59
Shelf of the North-western Black Sea		3.55	60
Shelf of the northern Mexico Gulf	-3.1-15.1	6.1	61
Shelf of the South China Sea		1.4	62
The Tokyo Bay, Japan	1.4-153.6	28.8	63
The Jiaozhou Bay, China		12	64
The Gulf Coast, USA	0-7.2	2.4	65

Based on these data, the median water-air N_2O flux in estuarine waters is about 21.6 μmol m^{-2} d^{-1}, while the median water-air N_2O flux in continental shelves is approximately 3.1 μmol m^{-2} d^{-1}. Locations of these study sites are shown in Supplementary Fig. 6.

Supplementary Table 5b. Estimated water-air N_2O flux from estuarine waters and coastal continental shelves.

	Area (km^2)	N_2O flux (μmol m^{-2} d^{-1})	N_2O flux (Tg N_2O-N yr^{-1})
Estuarine waters	1.025×10^6	21.6	0.23
Coastal continental shelves	2.72×10^7	3.1	0.86
Total			1.09

Supplementary Table 6. Carbonate chemistry of the CO_2 effect vs. pH effect experiments.

Treatment	pH	DIC (μmol kg^{-1})	Alkalinity (μmol kg^{-1})	pCO_2 (μatm)
400 μatm CO_2-pH 8.1	8.11 ± 0.02	2351 ± 18	2729 ± 8	388 ± 18
400 μatm CO_2-pH 7.8	7.81 ± 0.02	1119 ± 9	1238 ± 14	396 ± 12
800 μatm CO_2-pH 7.8	7.80 ± 0.03	2230 ± 17	2406 ± 14	796 ± 51
800 μatm CO_2-pH 8.1	8.11 ± 0.01	4692 ± 43	5332 ± 55	787 ± 7

Data represent mean ± SD (n=3 biologically independent samples).

Supplementary Table 7. Sequencing read statistics of the metatranscriptomic libraries of the enrichment cultures.

		Raw reads	Clean reads[a]	Seqs[b]	ORFs[c]
Nitrifying enrichment culture	Ambient control	86,253,870	86,086,432	323,127	293,847
	Acidified treatment	68,235,060	68,075,200	345,240	307,202
AOA enrichment culture	Ambient control	71,361,114	70,466,072	241406	183,546
	Acidified treatment	70,669,080	68,538,790	246398	190,893

[a]Clean reads represent the reads which were cleaned and trimmed (those shorter than 50 bp, containing ambiguous bases, and of low quality were removed).
[b]Seqs represent the *de novo* assembly results (contigs less than 200 base pairs in length were removed for the nitrifying enrichment culture and less than 300 base pairs in length were removed for the AOA enrichment culture).
[c]ORFs indicate the predicted genes (ORFs less than 100 base pairs in length were removed).

Supplementary Table 8. Definitions of the abbreviations in Figs. 4 and 5.

Abbreviation	Definition	Abbreviation	Definition
amo	ammonia monooxygenase	NXOR	putative nitroxyl oxidoreductase
hao	hydroxylamine dehydrogenase	NH_2OH	Hydroxylamine reductase
nxr	nitrite oxidoreductase	ACO	ATP-citrate lyase
nirK	nitrite reductase (NO-forming)	ACN	aconitate hydratase
nor	nitric oxide reductase	ACK	acetyl-CoA C-acetyltransferase
Cyt554	cytochrome c554	HBD	3-hydroxybutyryl-CoA dehydrogenase
mCyt552	cytochrome cm552	4HCD	4-hydroxybutyryl-CoA dehydratase
Cyt551	cytochrome c551	HBCS	4-hydroxybutyryl-CoA synthetase
Cyt552	cytochrome c552	MCM	methylmalonyl-CoA mutase
Cyt553	cytochrome c553	CCH	3-hydroxybutyryl-CoA dehydratase
Q	ubiquinone	HPCS	3-hydroxypropionyl-CoA synthetase
RBCL	ribulose-bisphosphate carboxylase large chain	MSR	malonic semialdehyde reductase
PGK	phosphoglycerate kinase	ictB	inorganic carbon (HCO_3^-) transporter
GAP	glyceraldehyde 3-phosphate dehydrogenase	ABC.FEV	iron complex transport system
TKT	transketolase	pst	phosphate transport system
RPI	ribose 5-phosphate isomerase	cys	sulfate transport system
PRK	phosphoribulokinase	ABC.SS	simple sugar transport system
KOR	2-oxoglutarate/2-oxoacid ferredoxin oxidoreductas	urt	urea transport system
SUC	succinyl-CoA synthetase	ABC-2	ABC-2 type transport system
SDH	succinate dehydrogenase	nuo	NADH-quinone oxidoreductase
FUM	fumarate hydratase	ndh	NADH dehydrogenase
MDH	malate dehydrogenase	pet	ubiquinol-cytochrome c reductase
ccmL	carbon dioxide concentrating mechanism protein	qcrA	menaquinol-cytochrome c reductase
MCE	methylmalonyl-CoA epimerase	cco	cytochrome c oxidase cbb3-type
livK, liv	branched-chain amino acid transport system	cox	cytochrome c oxidase
ICD	isocitrate dehydrogenase	amt	ammonium transporter
AccABC	acetyl-CoA carboxylase	ABC.SN	NitT/TauT family transport system
ABC.PE	peptide/nickel transport system substrate-binding protein	ddp	peptide/nickel transport system ATP-binding protein
ATPVA-K	V/A-type H^+/Na^+-transporting ATPase subunit A-K	phn	phosphonate transport system
QRED	quinone reductase	znu	zinc transport system
QH_2	ubiquinol	pcy	plastocyanins
CuHAO	copper hydroxylamine oxidoreductase	atp	ATPase

Supplementary Table 9. Expression of genes based on qPCR method.

Gene	Ambient control (copies ml^{-1})	Acidified treatments (copies ml^{-1})	p-value
Bacterial amoA[a]	$3.1\times10^{6} \pm 2.2\times10^{5}$	$9.7\times10^{5} \pm 2.1\times10^{4}$	$P<0.01$
hao[a]	$9.3\times10^{5} \pm 1.5\times10^{4}$	$3.3\times10^{5} \pm 1.8\times10^{4}$	$P<0.01$
nxrA[a]	$2.1\times10^{7} \pm 4.2\times10^{5}$	$4.0\times10^{6} \pm 3.1\times10^{5}$	$P<0.01$
nxrB[a]	$4.7\times10^{6} \pm 2.7\times10^{5}$	$1.4\times10^{6} \pm 1.1\times10^{5}$	$P<0.01$
nirK[a]	$1.4\times10^{6} \pm 1.2\times10^{5}$	$6.6\times10^{5} \pm 2.3\times10^{4}$	$P<0.01$
norB[a]	$3.7\times10^{3} \pm 8.6\times10^{2}$	$7.7\times10^{3} \pm 1.3\times10^{3}$	$P<0.05$
norC[a]	$2.8\times10^{3} \pm 5.4\times10^{2}$	$1.9\times10^{4} \pm 3.2\times10^{3}$	$P<0.05$
Archaeal amoA[b]	$6.2\times10^{5} \pm 3.4\times10^{4}$	$4.5\times10^{5} \pm 1.1\times10^{4}$	$P<0.05$

[a]Gene expression of the bacterial nitrifier enrichment culture, [b]gene expression of the AOA enrichment culture.
Data show mean value ± SD (n=3 biologically independent samples).
Statistical analyses were conducted via one-way analysis of variance (ANOVA) followed by Tukey's honestly test.

Supplementary Table 10. Detailed information for the primers and qPCR protocols used in the study.

Target gene	Primers	Sequence (5'-3')	Refs	qPCR conditions
Archaeal *amoA* gene	Arch-amoAF<br>Arch-amoAR	STAATGGTCTGGCTTAGACG<br>GCGGCCATCCATCTGTATGT	66	50°C for 2 min, 95 °C for 10 min, 45 × [95 °C for 30 s, 56 °C for 45 s, 72 °C for 50 s]
Bacterial *amoA* gene	amoA-1F<br>amoA-2R	GGGGTTTCTACTGGTGGT<br>CCCCTCKGSAAAGCCTTCTTC	67	50°C for 2 min, 95°C for 10 min, 45 × [95°C for 30 s, 58°C for 40 s, 72°C for 60 s]
Comammox clade A *amoA* gene	A378F<br>C616R	TGGTGGTGGTGGTCNAAYTAT<br>ATCATCCGRATGTACTCHGG	68	50°C for 2 min, 95°C for 10 min, 45 × [95°C for 15 s, 56.7°C for 30 s, 72°C for 45 s]
Comammox clade B *amoA* gene	comaB-244F<br>comaB-659R	TAYTTCTGGACRTTYTA<br>ARATCCARACDGTGT	69	50°C for 2 min, 95 °C for 10 min, 45 × [95 °C for 30 s, 52 °C for 45 s, 72 °C for 60 s]
Nitrospira 16S rRNA gene	Nspra-675f<br>Nspra-746r	GCGGTGAAATGCGTAGAKATCG<br>TCAGCGTCAGRWAYGTTCCAGAG	70	50°C for 2 min, 95°C for 10 min, 45 × [94°C for 30 s, 64°C for 30 s, 72°C for 60 s]
hao gene	Forward<br>Reverse	AGCCGATTCCGTTGAGTA<br>CTTGCCTTCTTGTCTAATGC	This study	50°C for 2 min, 95°C for 10 min, 45 × [95°C for 30 s, 58°C for 45 s, 72°C for 50 s]
nxrA gene	Forward<br>Reverse	CGATGTTGTGGCGGATTA<br>GCGTTAATGGTCTGATGGA	This study	50°C for 2 min, 95°C for 10 min, 45 × [95°C for 30 s, 56°C for 45 s, 72°C for 50 s]
nxrB gene	Forward<br>Reverse	ATGCCTGAAGTATATAACTG<br>GTCGTAGAACTGCGGATA	This study	50°C for 2 min, 95°C for 10 min, 45 × [95°C for 30 s, 58°C for 45 s, 72°C for 50 s]
nirK gene	Forward<br>Reverse	CTCTTCTGCTAACCGTCAA<br>ACTCGCCTTCAACATTCAT	This study	50°C for 2 min, 95°C for 10 min, 45 × [95°C for 30 s, 62°C for 45 s, 72°C for 50 s]
norB gene	Forward<br>Reverse	GATTCAGACGCATCTTCAAC<br>AGCAAGGCACCAATTAGAA	This study	50°C for 2 min, 95°C for 10 min, 45 × [95°C for 30 s, 56°C for 45 s, 72°C for 50 s]
norC gene	Forward<br>Reverse	GGAGGGATGATGGCAGAA<br>CGTTGATTCGTCCGTTGA	This study	50°C for 2 min, 95°C for 10 min, 45 × [95°C for 30 s, 56°C for 45 s, 72°C for 50 s]

All tests were performed in triplicate with amplification efficiencies between 90 and 110%. The correlation coefficients (R^2) were all >0.99. The primers designed in this study were based on the metatranscriptomic sequences, using Primer Premier 6.0 software. The abundance of comammox *amoA* gene was equal to that of comammox clade A *amoA* gene, as comammox clade B *amoA* gene was not detected.

Supplementary References:

1. Zheng, Y. L. et al. Effects of silver nanoparticles on nitrification and associated nitrous oxide production in aquatic environments. *Sci. Adv.* **3**, e1603229 (2017).

2. Takai, K. & Horikoshi, K. Rapid detection and quantification of members of the archaeal community by quantitative PCR using fluorogenic probes. *Appl. Environ. Microbiol.* **66**, 5066-5072 (2000).

3. Parada, A. E., Needham, D. M. & Fuhrman, J. A. Every base matters: assessing small subunit rRNA primers for marine microbiomes with mock communities, time series and global field samples. *Environ. Microbiol.* **18**, 1403-1414 (2016).

4. Apprill, A., McNally, S. P., Parsons, R. & Weber, L. Minor revision to V4 region SSU rRNA 806R gene primer greatly increases detection of SAR11 bacterioplankton. *Aquat. Microb. Ecol.* **75**, 129-137 (2015).

5. Caporaso, J. G. et al. QIIME allows analysis of high-throughput community sequencing data. *Nat. Methods* **7**, 335-336 (2010).

6. Edgar, R. C., Haas, B. J., Clemente, J. C., Quince, C. & Knight, R. UCHIME improves sensitivity and speed of chimera detection. *Bioinformatics* **27**, 2194-2200 (2011).

7. Cole, J. R. et al. Ribosomal Database Project: data and tools for high throughput rRNA analysis. *Nucleic Acids Res.* **42**, D633-D642 (2014).

8. Toyoda, S. et al. Characterization and production and consumption processes of N_2O emitted from temperate agricultural soils determined via isotopomer ratio analysis. *Glob. Biogeochem. Cycle*. **25**, GB2008 **(**2011).

9. Ostrom, N. E. & Ostrom, P. H. Mining the isotopic complexity of nitrous oxide: a review of challenges and opportunities. *Biogeochem.* **132**, 359-372 (2017).

10. Sutka, R. L. et al. Distinguishing nitrous oxide production from nitrification and denitrification on the basis of isotopomer abundances. *Appl. Environ. Microbiol.* **72**, 638-644 (2006).

11. Rathnayake, R. M. L. D. et al. Source identification of nitrous oxide on autotrophic partial nitrification in a granular sludge reactor. *Water Res.* **47**, 7078-7086 (2013).

12. Murray, R. H., Erler, D. V. &Eyre, B. D. Nitrous oxide fluxes in estuarine environments: response to global change. *Glob. Chang. Biol.* **21**, 3219-3245 (2015).

13. Jönsson, A., Gustafsson, O., Axelman, J. & Sundberg, H. Global accounting of PCBs in the continental shelf sediments. *Environ. Sci. Technol.* **37**, 245-255 (2003).

14. Seitzinger, S. P., Kroeze, C. & Styles, R. V. Global distribution of N_2O emissions from aquatic systems: Natural emissions and authropogenic effects. *Chemosphere* **2**, 267-279 (2000).

15. Cavicchioli, R. et al. Scientists' warning to humanity: microorganisms and climate change. *Nat. Rev. Microbiol.* **17**, 569-586 (2019).

16. Hong, H. Z. et al. The complex effects of ocean acidification on the prominent N_2-fixing cyanobacterium *Trichodesmium*. *Science* **356**, 527-531 (2017).

17. Beman, J. M. et al. Global declines in oceanic nitrification rates as a consequence of ocean acidification. *Proc. Natl Acad. Sci. U. S. A.* **108**, 208-213 (2011).

18. Ward, B. B. “Nitrification in marine systems” in *Nitrogen in the Marine Environment* (Elsevier, Amsterdam, 2008), pp. 199-261.
19. Badger, M. R. & Bek, E. J. Multiple Rubisco forms in proteobacteria: Their functional significance in relation to CO_2 acquisition by the CBB cycle. *J. Exp. Bot.* **59**, 1525-1541 (2008).
20. Scanes, E., Scanes, P. R. & Ross, P. M. Climate change rapidly warms and acidifies Australian estuaries. *Nat. Commun.* **11**, 1803 (2020).
21. Liu, X. H., Sun, D. Q., Huang, B. & Wang, J. X. Acidification and the factors in surface seawater of the East China Sea Coast. *Oceanologia et Limnologia Sinica* **48**, 398-405 (2017).
22. Shen, C. Q., Testa, J. M., Li, M. & Cai, W. J. Understanding anthropogenic impacts on pH and aragonite saturation in Chesapeake Bay: insights from a 30-year model study. *J. of Geophys. Res. Biogeosci.* **125**, e2019JG005620 (2020).
23. Lowe, A. T., Bos, J. & Ruesink, J. Ecosystem metabolism drives pH variability and modulates long-term ocean acidification in the Northeast Pacific coastal ocean. *Sci. Rep.* **9**, 963 (2019).
24. Carstensen, J., Chierici, M., Gustafsson, B. G. & Gustafsson, E. Long-term and seasonal trends in estuarine and coastal carbonate systems. *Global Biogeochem. Cycles* **32**, 497-513 (2018).
25. Robbins, L. L. & Lisle, J. T. Regional acidification trends in florida shellfish estuaries: a 20+ year look at pH, oxygen, temperature, and salinity. *Estuar. Coast.* **41**, 1268-1281 (2018).
26. Hu, X. P., Pollack, J. B., McCutcheon, M. R., Montagna, P. A. & Ouyang, Z. Long-term alkalinity decrease and acidification of estuaries in Northwestern Gulf of Mexico. *Environ. Sci. Technol.* **49**, 3401-3409 (2015).
27. Ishii, M. et al. Ocean acidification off the south coast of Japan: a result from time series observations of CO_2 parameters from 1994 to 2008. *J. Geophys. Res-Oceans* **116**, C06022 (2011).
28. Yao, H. M., Wang, J. J., Han, Y., Jiang, X. L. & Chen, J. S. Decadal acidification in a subtropical coastal area under chronic eutrophication, *Environ. Pollut.* **293**, 118487 (2022).
29. Rees, A. P., Brown, I. J., Jayakumar, A. & Ward, B. B. The inhibition of N_2O production by ocean acidification in cold temperate and polar waters. *Deep-sea Res. Pt. II* **127**, 93-101 (2016).
30. Breider, F. et al. Response of N_2O production rate to ocean acidification in the western North Pacific. *Nat. Clim. Change* **9**, 954-958 (2019).
31. Shiozaki, T. et al. Factors regulating nitrification in the Arctic Ocean: potential impact of sea ice reduction and ocean acidification. *Glob. Biogeochem. Cycles* **33**, 1085-1099 (2019).
32. Bange, H. W. et al. Seasonal study of methane and nitrous oxide in the coastal waters of the southern Baltic Sea. *Estuar. Coast. Shelf Sci.* **47**, 807-817 (1998).
33. Barnes, J. & Owens, N. J. P. Denitrification and nitrous oxide concentrations in the Humber Estuary, UK, and adjacent coastal zones. *Mar. Pollut. Bull.* **37**, 247-260 (1999).

34. de Angelis, M. A. & Gordon, L. I. Upwelling and river runoff as sources of dissolved nitrous oxide to the Alsea estuary, Oregon. *Estuar. Coast. Shelf Sci.* **20**, 375-386 (1985).
35. de Wilde, H. P. J. & de Bie, M. J. M. Nitrous oxide in the Schelde estuary: production by nitrification and emission to the atmosphere. *Mar. Chem.* **69**, 203-216 (2000).
36. Garnier, J., Cébron, A., Tallec, G., Billen, G. & Sebilo, M. A Martinez, Nitrogen behaviour and nitrous oxide emission in the tidal Seine River estuary (France) as influenced by human activities in the upstream watershed. *Biogeochemistry* **77**, 305-326 (2006).
37. Goncalves, C., Brogueira, M. J. & Camões, M. F. Seasonal and tidal influence on the variability of nitrous oxide in the Tagus estuary, Portugal. *Sci. Mar.* **74**, 57-66 (2010).
38. Jensen, H. B., Jørgensen, K. S. & Sørensen, J. Diurnal variation of nitrogen cycling in coastal, marine sediments. *Mar. Biol.* **83**, 177-183 (1984).
39. Rao, G. D. & Sarma, V. V. S. S. Contribution of N_2O emissions to the atmosphere from Indian monsoonal estuaries. *Tellus B* **65**, 19660 (2013).
40. LaMontagne, M. G., Duran, R. & Valiela, I. Nitrous oxide sources and sinks in coastal aquifers and coupled estuarine receiving waters. *Sci. Total Environ.* **309**, 139-149 (2003).
41. Law, C. S., Rees, A. P. & Owens, N. J. P. Nitrous oxide: estuarine sources and atmospheric flux. *Estuar. Coast. Shelf Sci.* **35**, 301-314 (1992).
42. Leip, A. Nitrous oxide (N_2O) emissions from a coastal catchment in the delta of the Po river: measurements and modeling of fluxes from a Mediterranean lagoon and agricultural soils. PhD thesis, University of Bayreuth, Bayreuth, Germany (2000).
43. Musenze, R. S., Werner, U., Grinham, A., Udy, J. & Yuan, Z. G. Methane and nitrous oxide emissions from a subtropical estuary (the Brisbane River estuary, Australia). *Sci. Total Environ.* **472**, 719-729 (2014).
44. Robinson, A. D., Nedwell, D. B., Harrison, R. M. & Ogilvie, B. G. Hypernutrified estuaries as sources of N_2O emission to the atmosphere: the estuary of the River Colne, Essex, UK. *Mar. Ecol. Prog. Ser.* **164**, 59-71(1998).
45. Silvennoinen, H., Liikanen, A., Rintala, J. & Martikainen, P. J. Greenhouse gas fluxes from the eutrophic Temmesjoki River and its Estuary in the Liminganlahti Bay (the Baltic Sea). *Biogeochemistry* **90**, 193-208 (2008).
46. Wong, W. W. et al. Dynamics of groundwater-derived nitrate and nitrous oxide in a tidal estuary from radon mass balance modeling. *Limnol. Oceanogr.* **58**, 1689-1706 (2013).
47. Rajkumar, A. N., Barnes, J., Ramesh, R., Purvaja, R. & Upstill-Goddard, R. C. Methane and nitrous oxide fluxes in the polluted Adyar River and estuary, SE India. *Mar. Pollut. Bull.* **56**, 2043-2051 (2008).
48. Ma, L., Lin, H., Xie, X. B., Dai, M. H. & Zhang, Y. Major role of ammonia-oxidizing bacteria in N_2O production in the Pearl River Estuary. *Biogeosciences* **16**, 4765-4781 (2019).

49. Lessin, G. et al. Modeling the seasonality and controls of nitrous oxide emissions on the northwest European continental shelf. *J. Geophys. Res-Biogeo* **125**, e2019JG005613 (2020).
50. Codispoti, L. A. On the nitrous oxide flux from productive regions that contain low oxygen waters, in: Oceanography of the Indian Ocean. (Oxford Univ. Press, New York, 1992), pp. 271-284.
51. Cornejo, M., Farías, L. & Gallegos, M. Seasonal cycle of N_2O vertical distribution and air-sea fluxes over the continental shelf waters off central Chile (~36°S), *Prog. Oceanogr.* **75**, 383-395 (2007).
52. Wu, M. et al. Spatial variability and factors influencing the air-sea N_2O flux in the Bering Sea, Chukchi Sea and Chukchi Abyssal Plain. *Atmosphere* **8**, 65 (2017).
53. Capelle, D. W. & Tortell, P. D. Factors controlling methane and nitrous-oxide variability in the southern British Columbia coastal upwelling system. *Mar. Chem.* **179**, 56-67 (2016).
54. Zhan, L. Y. et al. Austral summer N_2O sink and source characteristics and their impact factors in Prydz Bay, Antarctica. *J. Geophys. Res-Oceans* **120**, 5836-5849 (2015).
55. Walter, S., Bange, H. W. & Wallace, D. W. R. Nitrous oxide in the surface layer of the tropical North Atlantic Ocean along a west to east transect. *Geophys. Res. Lett.* **31**, L23S07 (2004).
56. Bange, H. W. Nitrous oxide and methane in European coastal waters. *Estuar. Coast. Shelf Sci.* **70**, 361-374 (2006).
57. Ferrón, S., Ortega, T. & Forja, J. M. Nitrous oxide distribution in the north-eastern shelf of the Gulf of Cádiz (SW Iberian Peninsula). *Mar. Chem.* **119**, 22-32 (2010).
58. Sierra, A. et al. Distribution of N_2O in the eastern shelf of the Gulf of Cadiz (SW Iberian Peninsula). *Sci. Total Environ.* **593-594**, 796-808 (2017).
59. Bange, H. W., Rapsomanikis, S. & Andreae, M. O. The Aegean Sea as a source of atmospheric nitrous oxide and methane. *Mar. Chem.* **53**, 41-49 (1996).
60. Amouroux, D., Roberts, G., Rapsomanikis, S. & Andreae, M. O. Biogenic gas (CH_4, N_2O, DMS) emission to the atmosphere from near-shore and shelf waters of the northwestern Black Sea. *Estuar. Coast. Shelf Sci.* **54**, 575-587 (2002).
61. Kim, I. N. Estimating "mean-state" July (1985–2007) N_2O fluxes in the northern Gulf of Mexico hypoxic region: Variation, distribution, and implication. *Front. Mar. Sci.* **5**, 249 (2018).
62. Han, Y., Zhang, G. L., Zhao, Y. C. & Liu, S. M. Distributions and sea-to-air fluxes of nitrous oxide in the coastal and shelf waters of the northwestern South China Sea. *Estuar. Coast. Shelf Sci.* **133**, 32-44 (2013).
63. Hashimoto, S., Gojo, K., Hikota, S., Sendai, N. & Otsuki, A. Nitrous oxide emissions from coastal waters in Tokyo Bay. *Mar. Environ. Res.* **47**, 213-223 (1999).
64. Zhang, G. L., Zhang, J., Xu, J. & Zhang, F. Distributions, sources and atmospheric fluxes of nitrous oxide in Jiaozhou Bay. *Estuar. Coast. Shelf Sci.* **68**, 557-566 (2006).

65. Smith, C. J., DeLaune, R. D. & Jr, W. H. P. Nitrous oxide emission from Gulf Coast wetlands. *Geochim. Cosmochim. Ac.* **47**, 1805-1814 (1983).
66. Francis, C. A., Roberts, K. J., Beman, J. M., Santoro, A. E. & Oakley, B. B. Ubiquity and diversity of ammonia-oxidizing archaea in water columns and sediments of the ocean. *Proc. Natl. Acad. Sci.* **102**, 14683-14688 (2005).
67. Rotthauwe, J. H., Witzel, K. P. & Liesack, W. The ammonia monooxygenase structural gene amoA as a functional marker: Molecular fine-scale analysis of natural ammonia oxidizing populations. *Appl. Environ. Microbiol.* **63**, 4704-4712 (1997).
68. Xia, F. et al. Ubiquity and diversity of complete ammonia oxidizers (comammox). *Appl. Environ. Microbiol.* **84**, e01390-18 (2018).
69. Pjevac, P. et al. AmoA-targeted polymerase chain reaction primers for the specific detection and quantification of comammox *Nitrospira* in the environment. *Front. Microbiol.* **8**, 1508 (2017).
70. Graham, D. W. et al. Experimental demonstration of chaotic instability in biological nitrification. *ISME J.* **1**, 385-393 (2007).
