# Supplementary material for: Effects of acidification on nitrification and associated nitrous oxide emission in estuarine and coastal waters
Source: Nat Commun. 2023 Mar 13;14:1380. doi: 10.1038/s41467-023-37104-9 (PMC10011576; doi:10.1038/s41467-023-37104-9)
Supplement: Supplementary file 3 — Reporting Summary [file 41467_2023_37104_MOESM3_ESM.pdf]

nature portfolio

Corresponding author(s): Lijun Hou (ljhou@sklec.ecnu.edu.cn); Min Liu (mliu@geo.ecnu.edu.cn); Yanling Zheng (ylzheng@geo.ecnu.edu.cn);

Last updated by author(s): Feb 10, 2023

Reporting Summary

Nature Portfolio wishes to improve the reproducibility of the work that we publish. This form provides structure for consistency and transparency in reporting. For further information on Nature Portfolio policies, see our Editorial Policies and the Editorial Policy Checklist.

Statistics

For all statistical analyses, confirm that the following items are present in the figure legend, table legend, main text, or Methods section.

n/a	Confirmed	
☐	☒	The exact sample size (*n*) for each experimental group/condition, given as a discrete number and unit of measurement
☐	☒	A statement on whether measurements were taken from distinct samples or whether the same sample was measured repeatedly
☐	☒	The statistical test(s) used AND whether they are one- or two-sided<br>*Only common tests should be described solely by name; describe more complex techniques in the Methods section.*
☐	☒	A description of all covariates tested
☐	☒	A description of any assumptions or corrections, such as tests of normality and adjustment for multiple comparisons
☐	☒	A full description of the statistical parameters including central tendency (e.g. means) or other basic estimates (e.g. regression coefficient) AND variation (e.g. standard deviation) or associated estimates of uncertainty (e.g. confidence intervals)
☐	☒	For null hypothesis testing, the test statistic (e.g. *F*, *t*, *r*) with confidence intervals, effect sizes, degrees of freedom and *P* value noted<br>*Give P values as exact values whenever suitable.*
☒	☐	For Bayesian analysis, information on the choice of priors and Markov chain Monte Carlo settings
☒	☐	For hierarchical and complex designs, identification of the appropriate level for tests and full reporting of outcomes
☒	☐	Estimates of effect sizes (e.g. Cohen's *d*, Pearson's *r*), indicating how they were calculated

Our web collection on statistics for biologists contains articles on many of the points above.

Software and code

Policy information about availability of computer code

Data collection	No software was used.
Data analysis	Data was analyzed using the following published softwares:<br>- FastQC v 0.11.4<br>- SeqPrep (https://github.com/jstjohn/SeqPrep) v 1.2<br>- Sickle (https://github.com/najoshi/sickle) v 1.33<br>- SortMeRNA v 2.1<br>- Trinity v 2.8.4<br>- MetaGeneMark v. 3.38<br>- BLAST v 2.2.28+<br>- CD-HIT v 4.6.8<br>- kallisto v 0.46.1<br>- IBM SPSS Statistics v 19.0<br>- IQ-TREE v 1.6.12<br>- iTOL v 6.6<br>- Qiime v 1.9.0<br>- Uchime algorithm v 4.2

Databases used to analyze data:
- nr (ftp://ftp.ncbi.nlm.nih.gov/)
- KEGG (https://www.genome.jp/kegg/)

Data Visualization:
- Origin v 2022b
- CorelDraw v 2019
- ArcGIS v10.2

For manuscripts utilizing custom algorithms or software that are central to the research but not yet described in published literature, software must be made available to editors and reviewers. We strongly encourage code deposition in a community repository (e.g. GitHub). See the Nature Portfolio guidelines for submitting code & software for further information.

Data

Policy information about availability of data

All manuscripts must include a data availability statement. This statement should provide the following information, where applicable:

- Accession codes, unique identifiers, or web links for publicly available datasets
- A description of any restrictions on data availability
- For clinical datasets or third party data, please ensure that the statement adheres to our policy

All sequence data and sample information are available at National Center for Biotechnology Information (NCBI) Sequence Read Archive (SRA) database under BioProject accession numbers PRJNA876082 [https://www.ncbi.nlm.nih.gov/bioproject/?term=PRJNA876082]. All data needed to evaluate the conclusions in the paper are present in the paper and/or the Supplementary Materials. Source data are provided with this paper.

Human research participants

Policy information about studies involving human research participants and Sex and Gender in Research.

Reporting on sex and gender	This item is not relevant to our study.
Population characteristics	This item is not relevant to our study.
Recruitment	This item is not relevant to our study.
Ethics oversight	This item is not relevant to our study.

Note that full information on the approval of the study protocol must also be provided in the manuscript.

Field-specific reporting

Please select the one below that is the best fit for your research. If you are not sure, read the appropriate sections before making your selection.

☐ Life sciences ☐ Behavioural & social sciences ☒ Ecological, evolutionary & environmental sciences

For a reference copy of the document with all sections, see nature.com/documents/nr-reporting-summary-flat.pdf

Ecological, evolutionary & environmental sciences study design

All studies must disclose on these points even when the disclosure is negative.

Study description	This study examined the responses of nitrification rate and associated N2O emission to aquatic acidification (pH reduced by 0.10-1.05) in estuarine and coastal waters. Manipulation experiments were also conducted to decouple the individual effects of elevated pCO2 and reduced pH, based on four groups of simulation systems: (a) 400 µatm/pH 8.1 (the ambient control group), (b) 400 µatm/pH 7.8 (reduction of pH only, maintaining pCO2 at the ambient level), (c) 800 µatm/pH 7.8 (the acidification group), (d) 800 µatm/pH 8.1 (increase of pCO2 only, maintaining pH at the ambient level). Metatranscriptomes were further analyzed to elucidate the metabolic response of nitrifying microbes by tracking the expression of acidification responsive genes. There were 3 replicates for each treatment.
Research sample	According to the geographic location, nitrification rates, nutrient concentration, and microbial communities, six representative sampling sites (Yz1 to Yz6) were selected along the Yangtze estuarine gradient from the estuary mouth to its adjacent coastal area. Near-bottom waters were collected from these sites during the March cruise of National Natural Science Foundation of China (NSFC) in 2020.
Sampling strategy	Near-bottom water samples were collected during the March cruise of National Natural Science Foundation of China (NSFC) in 2020, using Niskin-X bottles mounted on a conductivity-temperature-depth (CTD) profiler (Sea-Bird 911 plus). Water depth, salinity, pH and DO were recorded with CTD profiler, pH meter (Thermo Orion 3-Star) and Winkler's method. Part of the water from each site was

	preserved in dark at 4 °C for subsequent acidification experiments. Meanwhile, known amounts of subsamples were immediately filtered with 0.22 µm pore-size sterile filters (Waterman), and the filtrates were preserved for nutrient analyses while the membranes were carefully preserved under -20 °C for later DNA extraction. An extra amount of subsample from site Yz3 was also preserved at 4 ° C in dark for later long-term manipulation experiments.
Data collection	Metatranscriptomic cDNA libraries were constructed with the TruSeq RNA Sample Prep Kit (Illumina) and sequenced on an Illumina HiSeq4000 platform. Concentrations of CO2 and N2O were monitored by gas chromatography (GC-2014, Shimadzu, Kyoto, Japan). N2O isotope ratios (m/z = 44, 45, 46) were analyzed on isotope ratio mass spectrometry (IRMS, Delta V Advantage, Thermo Fisher Scientific, Bremen, Germany). Natural N2O isotopic signatures (δ15Nbulk, δ15Nα, and δ18O) for revealing N2O production pathways were analyzed on IRMS (Delta V Plus, Thermo Fisher Scientific, Bremen, Germany). Concentrations of NH4+, NO3− and NO2− were measured colorimetrically using a continuous-flow nutrient analyzer (Skalar SANplus, Skalar Analytical BV, Breda, The Netherlands) with detection limits of 0.3 µM for NH4+-N and 0.05 µM for NO2−-N and NO3−-N. DIC concentration was analyzed by acidification and subsequent quantification of released CO2 (Carbon coulometer, UIC-INC, America). J.Z., Y.L.Z., L.J.H., Z.R.A., F.Y.C., B.L.L., L.W., and L.Q. performed the research. J.Z., Y.L.Z., L.J.H., H.P.D., P.H., G.Y.Y., X.L., Y.Y., X.F.L., D.Z.G., Y.L., Z.F.L., R.B., and M.L. analyzed the data.
Timing and spatial scale	Six representative sampling sites (Yz1 to Yz6) were selected along the Yangtze estuarine gradient from the estuary mouth to its adjacent coastal area. Samples were collected during the March cruise of National Natural Science Foundation of China (NSFC) in 2020. Location of sampling collection was based on the geographic location, nitrification rates, nutrient concentration, and microbial communities. Among them, Yz3 was further selected for long-term acidification experiments. Yz3 was chosen because it was co-dominated by ammonia-oxidizing archaea (AOA) and ammonia-oxidizing bacteria (AOB).
Data exclusions	No data were excluded from the analysis in this study.
Reproducibility	The experiments were conducted in triplicate and the results were highly robust. The effects of acidification on nitrification was reproduced at all the six sampling sites.
Randomization	Each water sample collected in situ was mixed then randomly allocated into different groups for manipulation experiments.
Blinding	We were subjected to collecting a tremendous number of samples from multiple treatments. Therefore, we had to label treatments and samples clearly and accurately. Hence, it was not feasible for us to conduct measurements blindly.

Did the study involve field work? ☒ Yes ☐ No

Field work, collection and transport

Field conditions	Salinity of our sampling location ranges from 2.93 to 33.85. DO ranges from 8.30 to 9.86. Nitrate concentration ranges from 8.6 to 100.5 µM. Ammonium concentration ranges 1.7 to 9.8 µM. pH ranges from 7.92 to 8.15. There was no rain at the time of sampling.
Location	Water samples were collected from the Yangtze Estuary and adjacent coastal areas, China (Yz1, 121°36'E, 31°22'N, depth: 11m; Yz2, 122°7'E, 31°3'N, depth: 15m; Yz3, 122°15'E, 31°0'N, depth: 9m; Yz4, 122°25'E, 30°54'N, depth: 18m; Yz5, 122°45'E, 30°49'N, depth: 30m; Yz6, 123°15'E, 30°39'E, depth: 61m).
Access & import/export	Water samples were collected during the March cruise of National Natural Science Foundation of China (NSFC) in 2020. The collection of estuarine and coastal water in this study did not involve sensitive or prohibited areas.
Disturbance	There wasn't any disturbance.

Reporting for specific materials, systems and methods

We require information from authors about some types of materials, experimental systems and methods used in many studies. Here, indicate whether each material, system or method listed is relevant to your study. If you are not sure if a list item applies to your research, read the appropriate section before selecting a response.

Materials & experimental systems

n/a	Involved in the study	
☒	☐	Antibodies
☒	☐	Eukaryotic cell lines
☒	☐	Palaeontology and archaeology
☒	☐	Animals and other organisms
☒	☐	Clinical data
☒	☐	Dual use research of concern

Methods

n/a	Involved in the study	
☒	☐	ChIP-seq
☒	☐	Flow cytometry
☒	☐	MRI-based neuroimaging
